# A phase II study of weekly docetaxel and cisplatin plus oral tegafur/uracil and leucovorin as first-line chemotherapy in patients with locally advanced or metastatic gastric cancer

**DOI:** 10.1038/sj.bjc.6605928

**Published:** 2010-10-05

**Authors:** C-P Li, J-S Chen, L-T Chen, C-J Yen, K-D Lee, W-P Su, P-C Lin, C-H Lu, H-J Tsai, Y Chao

**Affiliations:** 1 Division of Gastroenterology, Department of Medicine, Taipei Veterans General Hospital, No. 201, Sec. 2, Shih-Pai Road, Taipei 11217, Taiwan; 2 National Yang-Ming University School of Medicine, Taipei, Taiwan; 3 Division of Hematology-Oncology, Department of Internal Medicine, Chang Gung Memorial Hospital and Chang Gung University, Taoyuan County, Taiwan; 4 Division of Cancer Research, National Health Research Institutes, Miaoli County, Taiwan; 5 Department of Internal Medicine, Kaohsiung Medical University Hospital, Kaohsiung, Taiwan; 6 Division of Hematology/Oncology, Department of Internal Medicine, National Cheng Kung University Hospital, Tainan, Taiwan; 7 Department of Hematology, Chang Gung Memorial Hospital, Chiayi, Taiwan; 8 Cancer Center, Taipei Veterans General Hospital, No. 201, Sec. 2, Shih-Pai Road, Taipei 11217, Taiwan

**Keywords:** docetaxel, cisplatin, tegafur/uracil, leucovorin, gastric cancer

## Abstract

**Background:**

Docetaxel plus cisplatin and 5-fluorouracil has become a new standard for treating advanced gastric cancer. However, high rates of severe neutropenia limit its application. Modification of the regimen could be the solution to get similar activity but less myelosuppression.

**Methods:**

Patients with histologically confirmed, locally advanced, or recurrent/metastatic gastric adenocarcinoma without previous chemotherapy were enrolled. This regimen consisted of docetaxel (Tyxan, TTY, Taipei, Taiwan) 30-min infusion at a dose of 36 mg m^−2^, followed by cisplatin 30 mg m^−2^ infusion over 1 h on days 1 and 8, and oral tegafur/uracil 300 mg m^−2^ per day plus leucovorin 90 mg per day on days 1–14, every 3 weeks. Tumour response was evaluated after every 2 cycles of treatment.

**Results:**

From August 2007 to March 2009, 45 patients were enrolled. The median age was 56 years (range: 22–75). Among the 40 patients evaluable for tumour response, one achieved a complete response, 22 had partial responses and 11 had stable disease. The overall response rates of the evaluable and intent-to-treat (ITT) populations were 58% (95% CI: 41–74%) and 53% (95% CI: 38–68%), respectively. The disease control rates in these populations were 85% (95% CI: 70–94%) and 82% (95% CI: 68–92%), respectively. In the ITT analysis, the median time to progression and overall survival were 6.8 and 13.9 months, respectively. Major grade 3–4 toxicities were neutropenia (51%), anaemia (22%), diarrhoea (16%), and infections (20%). No patient died of treatment-related toxicities.

**Conclusion:**

Concurrent weekly docetaxel and cisplatin plus oral tegafur/uracil and leucovorin are effective and well tolerated in the treatment of advanced gastric cancer.

Gastric cancer is one of the leading causes of cancer death worldwide (Roder, 2002). Although an early-stage disease can be treated with curative resection, most patients show locally advanced or metastatic disease on diagnosis. Furthermore, recurrent tumours are observed in up to 50% of the patients treated by curative intention using standardised surgical techniques (McNeer *et al*, 1951; Dupont *et al*, 1978). The prognosis is generally poor, with an overall 5-year survival of approximately 20% in most countries. The median survival time for patients who presented with advanced or metastatic diseases and had the best supportive care was only 3–5 months (Verdecchia *et al*, 2003).

With the introduction of novel drug administration schedules and the emergence of new chemotherapeutic agents, modern systemic chemotherapy can achieve 30–60% objective response rates in advanced gastric cancers (Schoffski, 2002). However, the overall survival (OS) remains consistently around 9–11 months, in the expenditure of significant treatment-related toxicities (Ohtsu, 2005). New treatments with better therapeutic indexes are very important for clinical management of these patients.

The non-overlapping toxicity profile of docetaxel, cisplatin, and oral fluoropyrimidine, as well as the observations of schedule-dependent synergism among docetaxel, cisplatin, and oral fluoropyrimidine in human gastric carcinoma xenografts (Maeda *et al*, 2004; Kodera *et al*, 2005), warrants such a combination to be evaluated in treating advanced gastric cancers. Recently, a multicenter, open-label, randomised phase III trial (V325) was conducted to evaluate the safety and efficacy of docetaxel for treating patients with advanced gastric adenocarcinoma (Van Cutsem *et al*, 2006). The patients were randomised to receive either the investigational arm of the triplet combination of docetaxel, cisplatin, and 5-fluorouracil (5-FU) (DCF), or the reference standard regimen of cisplatin and 5-FU. Both overall response rate (37 *vs* 25%) and time to progression (TTP) (5.6 *vs* 3.7 months) favored the DCF arm over the CF arm. The median OS was also significantly longer for the DCF arm than the CF arm (9.2 *vs* 8.6 months). These findings represent an important milestone in the treatment of patients with advanced gastric cancer and show that DCF can become a new standard for treating advanced gastric cancer. However, grade 3 or 4 neutropenia occurred in 82% of patients on DCF, including 29% with complicated neutropenia. The high rates of severe myelosuppression in the V325 study could have been caused by (1) the 3-weekly regimen of docetaxel, (2) high-dose cisplatin, and (3) 5-day continuous infusion of 5-FU.

To overcome the significant myelosuppression, weekly docetaxel at a dose of 36 mg m^−2^ and cisplatin 30 mg m^−2^ was used to replace the 3-weekly regimen, based on the previous reports that a relatively lower dosage and a weekly regimen had similar efficacy and better tolerability than the standard 3-weekly regimen (Kunitoh *et al*, 1996; Hainsworth *et al*, 1998; Chen *et al*, 2002; Schuette *et al*, 2005). Oral tegafur/uracil was used to replace the 5-days continuous infusion of 5-FU. This phase II study of combination chemotherapy of weekly docetaxel, cisplatin, and oral tegafur/uracil and leucovorin was conducted to determine if this regimen could achieve activity similar to the original schedule in the V325 study and be more tolerable for Asian patients with advanced gastric adenocarcinoma.

## Patients and methods

### Study design and patients

This was a prospective, multi-center, phase II clinical trial of docetaxel and cisplatin plus oral tegafur/uracil and leucovorin in patients with advanced gastric cancer. The primary objective was to determine the objective response rate in patients with advanced gastric cancer. Secondary objectives included TTP, duration of objective response, OS, and safety profiles.

Eligibility criteria of patients included (1) pathologically confirmed, locally advanced (unresectable), recurrent or metastatic gastric adenocarcinoma, including gastroesophageal junction tumours; (2) measurable disease by imaging studies; (3) no previous chemotherapy except for postoperative adjuvant chemotherapy received more than 6 months before entry into the study; (4) ECOG (Eastern Cooperative Oncology Group) performance status ⩽2; (5) age between 20 and 75 years; and (6) adequate hepatic, renal, and bone marrow functions, including aspartate aminotransferase/alanine aminotransferase ⩽2.5 times the upper limit of normal) (ULN) (⩽5.0 times the ULN if hepatic metastasis), alkaline phosphatase ⩽2.5 times the ULN (⩽5.0 times the ULN if bone metastasis), total bilirubin ⩽1.5 times the ULN, creatinine ⩽1.0 times the ULN, haemoglobin ⩾9 g per 100 ml, absolute neutrophil count (ANC) ⩾1500 per *μ*l, and platelets ⩾100 000 per *μ*l.

Exclusion criteria included (1) pre-existing peripheral neuropathy; (2) pregnancy, breastfeeding, or woman of child-bearing potential without adequate contraception; (3) concurrent or previous malignancy; (4) central nervous system metastases; (5) uncontrolled infection; (6) concurrent treatments that might interfere with the study evaluation; and (7) inability to take the study medication orally.

This study was approved by the ethics committees of all participating institutes and signed informed consent was obtained from all patients.

### Chemotherapy protocol

Docetaxel (Tyxan, TTY Biopharm, Taipei, Taiwan) 30-min infusion at a dose of 36 mg m^−2^, followed by cisplatin 30 mg m^−2^ infusion over 1 h on days 1 and 8, and oral tegafur/uracil (UFUR) 300 mg m^−2^ per day plus leucovorin (Folina) 90 mg per day on days 1–14, every 21 days, was administered. To reduce the risk of hypersensitivity reactions to docetaxel, all patients were pre-medicated with oral dexamethasone 8 mg twice daily for 3 days starting 1 day before docetaxel, or intravenous dexamethasone 10 mg 30 min before receiving docetaxel.

The total dose of cisplatin was dissolved in 250 ml of sterile saline and infused over a 1-hour period. Pretreatment hydration with 1–2 l of fluids infused before cisplatin dose was recommended. The total daily dose of tegafur/uracil was determined and rounded to the nearest 100 mg and divided into three doses given 8 h apart. If the total number of tablets could not be evenly divided, the highest dose was given in the morning and the lower doses in the afternoon or evening. Cycles were repeated every 3 weeks or on recovery from toxicities to baseline or grade 1 (except for alopecia and anaemia).

### Dose modification

Dose modifications were based on haematologic and non-haematologic toxicities. Haematology test was performed every week to monitor haematologic recovery. Chemotherapy doses were reduced or treatment was delayed for no more than 2 weeks to allow patients to recover from toxicities. The dose of each study drug was reduced stepwise. Level-1 was docetaxel 30 mg m^−2^, cisplatin 25 mg m^−2^, tegafur/uracil 200 mg m^−2^, and leucovorin 90 mg per day. Level-2 was docetaxel 25 mg m^−2^ and cisplatin 20 mg m^−2^, whereas tegafur/uracil and leucovorin were withdrawn. A maximum of two dose level reductions were allowed per patient and any patient who required more was excluded from the treatment protocol.

If a haematology test within 72 h before the next cycle indicated a neutrophil count <1500 per mm^3^ or a platelet count <100 000 per mm^3^, the next cycle was delayed until haematologic recovery. At haematologic nadir, if the neutrophil count was <1000 mm^3^ or platelets was <50 000 mm^3^, the next dose was reduced by one level on day 1 of a new cycle. If the neutrophil count was <1000 mm^3^ or the platelet count was <50 000 mm^3^ on day 8 within a cycle, the chemotherapy was omitted. The prophylactic use of recombinant granulocyte colony-stimulating factor (G-CSF) was not permitted. However, if a patient develops grade 4 neutropenia (ANC <500 per mm^3^), the use of recombinant G-CSF was allowed for subsequent cycles.

Chemotherapy administration was resumed at the same dose level during the cycle on recovery from non-haematologic toxicities to the baseline or grade 1 (except for alopecia and anaemia). If such recovery was not achieved before day 8 from the scheduled date, the chemotherapy dose was skipped on day 8. The next dose was reduced by one level. If a patient developed grade 1 or 2 nephrotoxicity (creatinine 1.0–3.0 times the ULN), cisplatin was omitted, and then reduced one dose level in the next dose on recovery to grade 1. If more than two reductions of dose level were needed, the patient was withdrawn from the study. If grade 3 or 4 nephrotoxicity (>3.0 times the ULN) occurred, the patient was withdrawn from the study. If diarrhoea greater than grade 2 occurred, tegafur/uracil and leucovorin were reduced one dose level plus the patient was given appropriate treatment for diarrhoea. If it reappeared, then the patient was withdrawn from the study. When toxicity subsided to baseline or less than grade 1, administration was resumed. Therapy was withheld until recovery for a maximum of 2 weeks; otherwise, the patient was withdrawn from the study. Treatment was not continued after treatment cycle day 14 regardless of the number of days the drug was interrupted. Treatment was administered until disease progression, intolerable toxicity, or consent withdrawal.

### Evaluation of efficacy and toxicities

Evaluations before chemotherapy included obtaining patient's medical history, physical examination, complete blood count, blood chemistry, chest X-ray, and abdominal computed tomography (CT). After starting the protocol treatment, complete blood count was examined weekly and blood chemistry every 3 weeks. Detailed medical history, physical examinations, and treatment-related toxicities were recorded weekly. Tumour size was measured by imaging studies every 6 weeks. Tumour response was evaluated according to the Response Evaluation Criteria in Solid Tumors (RECIST) criteria (Therasse *et al*, 2000). All patients with tumour responses (complete or partial response) received a confirmatory scan 4 weeks following the initial documentation.

Toxicities were graded, from the time of study entry through 30 days after the last dose of study medication was administered, according to the National Cancer Institute-Common Terminology Criteria for Adverse Events (v3.0) (Trotti *et al*, 2003).

### Statistical methods

The treatment programme was designed to reject a response rate of <25% (P0) and to provide a statistical power of 0.80 in assessing the activity of the regimen as 50% (P1) with an *α*-error of 0.05. A total of 37 patients were therefore required. Time to progression was defined as the duration from the date of starting protocol treatment to the date of disease progression. Data of patients who were lost to follow-up before progression were censored at the date of their last CT scan. Overall survival represented the duration from the date of starting protocol treatment to the date of death from any cause. Survival was estimated by the Kaplan–Meier analysis. All statistics were two-sided and performed using SAS software (version 9.1.3, SAS Inc., Cary, NC, USA).

## Results

### Patients and treatment

Between August 2007 and March 2009, 45 patients from five medical centres were enrolled in the study. Their clinicopathological characteristics are listed in Table 1. A total of 271 (median: 6; range: 1–21) cycles of chemotherapy were given. The median relative dose intensity was 82% (range: 46–100%) for docetaxel, 80% (range: 46–100%) for cisplatin, 95% (range: 54–100%) for tegafur/uracil, and 95% (range: 54–100%) for leucovorin. In total, 62% of the patients received more than 80% of the intended doses of docetaxel, 53% received more than 80% of the intended doses of cisplatin, and 89% received more than 80% of the intended doses of tegafur/uracil and leucovorin.

### Efficacy

Five patients were not evaluable for responses: three failed to return for tumour measurements, one was later found to have protocol violation, and one refused further chemotherapy after one treatment cycle. Among the 40 evaluable patients, the best tumour response was complete response in 1, partial response (PR) in 22, stable disease in 11, and progressive disease in 6. The overall response rates of the evaluable and intent-to-treat (ITT) populations were 58% (95% CI: 41–74%) and 53% (95% CI: 38–68%), respectively, whereas the disease control rate in according population was 85% (95% CI: 70–94%) and 82% (95% CI: 68–92%), respectively. The response rates of patients with ⩾80 and <80% of the scheduled docetaxel dose intensity were 58% (19 out of 33) and 57% (4 out of 7), respectively (*P*=1.0000). The response rates of patients with ⩾80 and <80% of scheduled cisplatin dose intensity were 62% (18 out of 29) and 45% (5 out of 11), respectively (*P*=0.7665). The response rates of patients with ⩾80 and <80% of scheduled tegafur/uracil dose intensity were 57% (20 out of 35) and 60% (3 out of 5), respectively (*P*=1.0000). Median time to tumour response was 1.8 (range: 1.2–6.4) months. Median duration of tumour response was 5.5 (range: 2.4–14.9) months. Of the 45 patients, 28 received second-line therapies. Among them, 19 patients received cisplatin plus 5-FU and leucovorin, 4 patients received oxaliplatin plus 5-FU and leucovorin, 1 patient received oxaliplatin plus tegafur/uracil and leucovorin, 1 patient received 5-FU and leucovorin, 1 patient received oxaliplatin and capecitabine, 1 patient received cisplatin plus 5-FU, leucovorin, and cetuximab, and 1 patient received everolimus.

**Figure 1:**
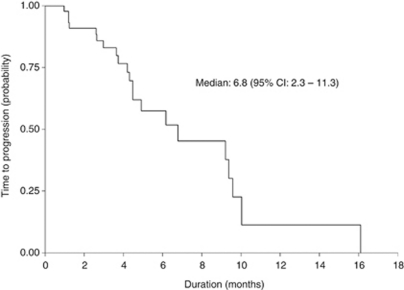
Time to progression of the 45 patients.

**Figure 2:**
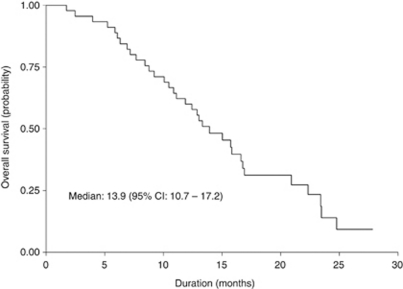
Overall survival of the 45 patients.

Median follow-up time was 13.3 (range: 1.7–27.8) months. Median TTP and OS were 6.8 (95% CI: 2.3–11.3) months and 13.9 (95% CI: 10.7–17.2) months, respectively. The Kaplan–Meier estimated TTP and OS curves are shown in Figures 1 and 2, respectively. The fraction of patients alive at 1 year was 60% and at 2 years was 7%.

### Toxicity

All patients were evaluated for toxicities (Table 2). The most common toxicity was neutropenia, with grades 3–4 neutropenia observed in 51% of the patients. Two patients developed neutropenic fever and recovered with appropriate therapy. Grade 3 anaemia was observed in 22% of the patients. Grades 3–4 diarrhoea developed in seven patients (16%). Grade 3 infections occurred in nine patients (20%), with one device-related infection, one nail infection, one wound infection, one urinary tract infection, and five pneumonia. A total of 36 (80%) patients had dose delays during treatment. Dose modification to level-1 was required in 19 (42%) patients, whereas level-2 dose modification was required in two patients (4%). Treatment-related toxicity resulted in treatment discontinuation in eight (18%) patients, which were associated with neutropenia in two, thrombocytopenia in two, pneumonia in two, anaemia in one and serum creatinine increased in one. There was no treatment-related mortality.

## Discussion

The V325 study showed that docetaxel in combination with cisplatin and 5-FU (DCF) could become a new standard therapy for the treatment of advanced gastric cancer (Van Cutsem *et al*, 2006). However, grade 3 or 4 neutropenia occurred in 82% of patients on DCF. The high incidence of neutropenia becomes a critical issue for clinical application of the DCF regimen. Recently, the superior therapeutic index of weekly docetaxel over the 3-weekly schedule has been demonstrated in gastric cancer patients (Ajani *et al*, 2005; Evans *et al*, 2007b; Roth *et al*, 2007; Wang *et al*, 2009). With comparable tumour response rates (37–48%) for advanced gastric cancer, grades 3–4 neutropenia of weekly docetaxel (33.3 mg m^−2^, on days 1, 8, and 15 every 4 weeks or 40 mg m^−2^, on day 1 and 8 every 3 weeks) was less common than that of 3-weekly docetaxel (75–85 mg m^−2^, on day 1 every 3 weeks) (<10–48 *vs* 57–86%). Weekly docetaxel and platinum plus either infusional 5-FU or oral fluoropyrimidine have become popular investigational combinations for advanced gastric cancers (Chen *et al*, 2002; Evans *et al*, 2007a, 2007b; Wang *et al*, 2009).

This is the first phase II study investigating weekly docetaxel and platinum plus oral fluoropyrimidine in treating advanced gastric cancer. The 58% overall response rate of the evaluable patients (53% on ITT analyses), median survival of 13.9 months and grades 3–4 neutropenia in 51% of participants are not inferior to the results of other phase II clinical trials with topoisomerase I inhibitor-, taxane-, or third-generation oral fluoropyrimidine-based (i.e., S-1 and capecitabine) doublet chemotherapy regimens (Schoffski, 2002; Ohtsu, 2005), in which the objective tumour response rate and median survival ranged from 40 to 76% and from 9 to 12.5 months, respectively. The median survival in this study was also comparable with those achieved in previous high-dose 5-FU and leucovorin-based studies in Taiwan (Hsu *et al*, 1997; Chen *et al*, 2002; Chao *et al*, 2004, 2006), and to the 6.1–12.0 months median survival of phase II or III studies of current ‘reference’ regimens for advanced gastric cancers, that is, ECF, FAMTX, ELF, FUP, FOLFOX-6, and DCF (Webb *et al*, 1997; Vanhoefer *et al*, 2000; De Vivo *et al*, 2000; Louvet *et al*, 2002; Ohtsu, 2005; Van Cutsem *et al*, 2006).

The overall response rates of evaluable and ITT populations were 58 and 53% in this study. Among other docetaxel-based studies, the tumour response rate ranged from 37 to 43% in chemo-naive gastric cancer patients (Ajani *et al*, 2005; Van Cutsem *et al*, 2006; Roth *et al*, 2007). The OS of 13.9 months, TTP of 6.8 months, and toxicity profiles (51% grades 3–4 neutropenia) of the current regimen are also comparable with those observed in previous docetaxel-based trials (OS 9.2–10.4 months, TTP 4.6–5.9 months, and 57–86% grades 3–4 neutropenia) (Ajani *et al*, 2005; Van Cutsem *et al*, 2006; Roth *et al*, 2007). Although this regimen was less toxic than other previously described combination therapies, the incidence of grade 4 neutropenia (18%) and treatment discontinuation due to treatment-related toxicity (18%) was still high. Further modification of the dosage of this regimen or addition of prophylactic G-CSF should be considered in the management of these patients. On the other hand, 76% of our patients had undergone gastrectomy previously, and the incidence of liver metastasis (20%) or peritoneal seeding (2%) was relatively low compared with that in other studies. A large proportion of recurrent disease and low tumour burden could have contributed to the good response rate and survival results in this study (Lee *et al*, 2007).

The advantages of oral fluoropyrimidine include the alleviation of the requirement and cost of central venous catheter implantation and the inconvenience of an infusion pump, which in turn may improve the quality of life of treated patients. In this study, we used tegafur/uracil replacing infusional 5-FU. Both tegafur/uracil and capecitabine are commonly used oral fluoropyrimidines and have similar antitumor activities (Twelves *et al*, 2005; Lembersky *et al*, 2006). Capecitabine has a higher rate of grades 3 and 4 hand foot syndrome, whereas tegafur/uracil has a higher rate of grades 3 and 4 diarrhoea (Twelves *et al*, 2005; Lembersky *et al*, 2006). This is consistent with our findings of no grades 3 and 4 hand foot syndrome, but occurrence of grade 3 diarrhoea in 13% of our patients.

Orditura *et al* (2006) reported that weekly docetaxel and capecitabine is not effective in the treatment of advanced gastric cancer. In that study, the dose of docetaxel was similar to that of our current study, whereas the dose of capecitabine was relatively low at 625 mg m^−2^ twice daily *per os* on days 5–18 repeated every 4 weeks, which may be inadequate for gastric cancer. Despite a low rate of myelosuppression, diarrhoea, and hand foot syndrome, the response rate was only 21%. In our study, the higher response rate could be due to adequate tegafur/uracil dosage (300 mg m^−2^ per day as recommended for gastric cancer). Moreover, there was a synergistic effect among cisplatin, tegafur/uracil, and docetaxel (Maeda *et al*, 2004; Kodera *et al*, 2005).

Recently, several novel targeted therapeutic agents such as inhibitors of epidermal growth factor receptor or of vascular endothelial growth factor, when in combination with chemotherapy, have shown promising activity against gastrointestinal cancers (Chong and Cunningham, 2005). The low-toxicity profiles of such agents also provide an excellent chance to improve the therapeutic index of this active and convenient regimen for advanced gastric cancer patients.

In conclusion, the combination of weekly docetaxel, cisplatin and oral tegafur/uracil, and leucovorin is an active, outpatient-based chemotherapy regimen with acceptable toxicities. Strategies to improve the therapeutic index of the current regimen, such as administration in combination with novel biological-targeted agents, should be further explored.

## Figures and Tables

**Table 1:** Clinicopathologic features of the patients

	**Patient number (%)**
Total patients	45
Age (years), median (range)	56 (22–75)
Sex: male/female	27/18
	
*ECOG performance*	
0	20 (44)
1	23 (51)
2	2 (4)
	
*Treatments for primary tumour*	
No previous therapy	9 (20)
Surgery only	27 (60)
Surgery+adjuvant chemotherapy	7 (16)
Radiotherapy	2 (4)
	
*Disease status*	
Locally advanced	4 (9)
Recurrence/metastasis	41 (91)
	
*Disease sites*	
Liver	9 (20)
Lymph nodes	34 (76)
Peritoneum	1 (2)
Gastrointestinal tract	25 (56)
Bone	2 (4)
Lung	0 (0)
Others	24 (53)

**Table 2:** Percentages of toxicity of the docetaxel, cisplatin, tegafur/uracil, and leucovorin regimen

	**Patients (*n*=45)**				**Cycles (*n*=271)**			
	**Grade**		**Grade**		**Grade**		**Grade**	
**Toxicity**	**1**	**2**	**3**	**4**	**1**	**2**	**3**	**4**
*Haematological*								
Neutropenia	4^a^	11	33	18	11^b^	13	17	6
Leukopenia	7	31	24	9	17	16	10	1
Thrombocytopenia	18	9	2	2	21	2	0.4	0.4
Febrile neutropenia^c^	0	0	4	0	0	0	1	0
Anaemia	2	58	22	0	4	27	5	0
								
*Gastrointestinal*								
Nausea	31	22	7	0	19	6	1	0
Vomiting	22	29	4	0	12	7	1	0
Diarrhoea	22	33	13	2	16	14	3	0
Stomatitis	13	22	7	0	5	11	4	0
Anorexia	24	24	4	0	18	6	8	0
Weight loss	20	16	2	0	10	6	0.4	0
								
Neurosensory	7	2	0	0	10	1	0	0
								
*Others*								
Hand and foot syndrome	4	4	0	0	3	5	0	0
Fever	16	7	0	0	6	1	0	0
Infections	11	16	20	0	5	5	7	0
Alopecia	36	9	0	0	31	5	0	0

a All numbers are percentages of the 45 patients.

b All numbers are percentages of the 271 cycles given.

c Febrile neutropenia was defined as fever of unknown origin without clinically or microbiologically documented infection with absolute neutrophil count <1.0 × 10^9^/l and fever ⩾38.5°C, according to the National Cancer Institute Common Terminology Criteria for Adverse Events 3.0.
